# Neuronal representation of stand and squat in the primary motor cortex of monkeys

**DOI:** 10.1186/s12993-015-0061-0

**Published:** 2015-04-09

**Authors:** Chaolin Ma, Xuan Ma, Hang Zhang, Jiang Xu, Jiping He

**Affiliations:** Center for Neuropsychiatric Disorders, Institute of Life Science, Nanchang University, Nanchang, 330031 China; Center for Neural Interface Design, School of Biological and Health Systems Engineering, Arizona State University, Tempe, AZ 85287 USA; Neural Interface & Rehabilitation Technology Research Center, Huazhong University of Science and Technology, Wuhan, 430074 China

**Keywords:** Monkeys, Single neuron recording, Topographical information, Lower limb movement, Neuroprosthetics

## Abstract

**Background:**

Determining neuronal topographical information in the cerebral cortex is of fundamental importance for developing neuroprosthetics. Significant progress has been achieved in decoding hand voluntary movement with cortical neuronal activity in nonhuman primates. However, there are few successful reports in scientific literature for decoding lower limb voluntary movement with the cortical neuronal firing. We once reported an experimental system, which consists of a specially designed chair, a visually guided stand and squat task training paradigm and an acute neuron recording setup. With this system, we can record high quality cortical neuron activity to investigate the correlation between these neuronal signals and stand/squat movement.

**Methods/results:**

In this research, we train two monkeys to perform the visually guided stand and squat task, and record neuronal activity in the vast areas targeted to M1 hind-limb region, at a distance of 1 mm. We find that 76.9% of recorded neurons (1230 out of 1598 neurons) showing task-firing modulation, including 294 (18.4%) during the pre-response window; 310 (19.4%) for standing up; 104 (6.5%) for the holding stand phase; and 205 (12.8%) during the sitting down. The distributions of different type neurons have a high degree of overlap. They are mainly ranged from +7.0 to 13 mm in the Posterior-Anterior dimension, and from +0.5 to 4.0 mm in Dosal-lateral dimension, very close to the midline, and just anterior of the central sulcus.

**Conclusions/significance:**

The present study examines the neuronal activity related to lower limb voluntary movements in M1 and find topographical information of various neurons tuned to different stages of the stand and squat task. This work may contribute to understanding the fundamental principles of neural control of lower limb movements. Especially, the topographical information suggests us where to implant the chronic microelectrode arrays to harvest the most quantity and highest quality neurons related to lower limb movements, which may accelerate to develop cortically controlled lower limb neuroprosthetics for spinal cord injury subjects.

## Background

Over the past two decades, numbers of researches have been reported in investigation of the relationship between the firing pattern of cortical neurons and the animal’s behavioural performance [1-12]. Using behavioural neurophysiological methods, significant progress has been achieved in the study of topographical information of the cerebral cortex [13-17]. These pioneering studies provide hopes and promises for the development of neuroprostheses for spinal cord/brain injured patients or people suffering from many neural degenerative diseases with limb dyskinesia.

Technical challenges to record cortical unit spikes in conscious behaviour monkey have been presented in the investigation of cortical control of lower limb. For example, microdrivable electrode recording technology requires the limitation of movement of the subject’s head [18]. It is the deficiency of this technology that limits its utility and using range. Quite a few of current research achievements in this area are focused on the upper limb movements [19-32]. For lower limb movements, there are few reports [33]. We have developed a novel experimental system for recording cortical neuronal signals related to stand and squat movement [34]. The system consists of a specially designed chair, a stand and squat task paradigm and a neuron recording setup with microdrivable electrodes. With this system, a monkey can perform visually guided stand and squat while the head is relatively stationary to allow accurate single cortical unit recording. The neuronal firing patterns, the kinematics and muscle activities were recorded simultaneously for analysing the cortical control of lower limb functions.

Determining neuronal topographical information in the cerebral cortex is of fundamental importance for developing neuroprosthetics. In this study, we trained two monkeys to perform a visually-cued stand and squat task and scanned the whole primary motor cortex at a distance of 1 mm, and recorded 1598 firing neurons. This work may contribute to the understanding of the fundamental principles of neural control of lower limb movement. By knowing how cortical neurons modulate their activities to initiate and control the standing and sitting movement we may eventually be able to develop cortically controlled neuroprosthetic systems for spinal cord injury patients.

Spinal cord injuries are particularly tragic for most of the victims are injured in the prime of life. Every year, there are over 350,000 people worldwide who are diagnosed with the tragically debilitating consequences of dysfunction in lower limb motor control, so it is obvious for the significance in searching for effective means of helping these people to regain ability to stand up and walk.

## Methods

### Subjects

Our data were collected from two male rhesus (*Macaca mulatta*) monkeys (Monkey H and Monkey V, 4–6 years old) to identify the cortical areas related to lower limb voluntary movements. The animal behavioural training and the actual experimental protocols had been described in detail in our previous paper [34]. The experiments were complied with NIH policy on Humane Care and Use of laboratory animals, and were approved by the institutional Animal Care and Use Committee, Arizona State University, USA.

### Experimental protocol

Each monkey was trained to perform a visually-cued stand and squat task using a well-designed primate chair. The chair was made of stainless steel and acrylic plastic, equipped with a movable pedal. While the head and body were restrained in the chair, the subject can still perform stand and sit movement, via pushing down the pedal with adjustable weight. The pedal was connected to the chair with two inner-set Axletrees, which made it possible to move up and down easily with negligible friction. When the subject wanted to stand up, the pedal can be pushed down to achieve the upright standing posture. When the subject intended to sit down and release the pushing force, the pedal was pulled up by the counter weight through the sliding axles, which were set on the back part of the chair.

**Figure 1:**
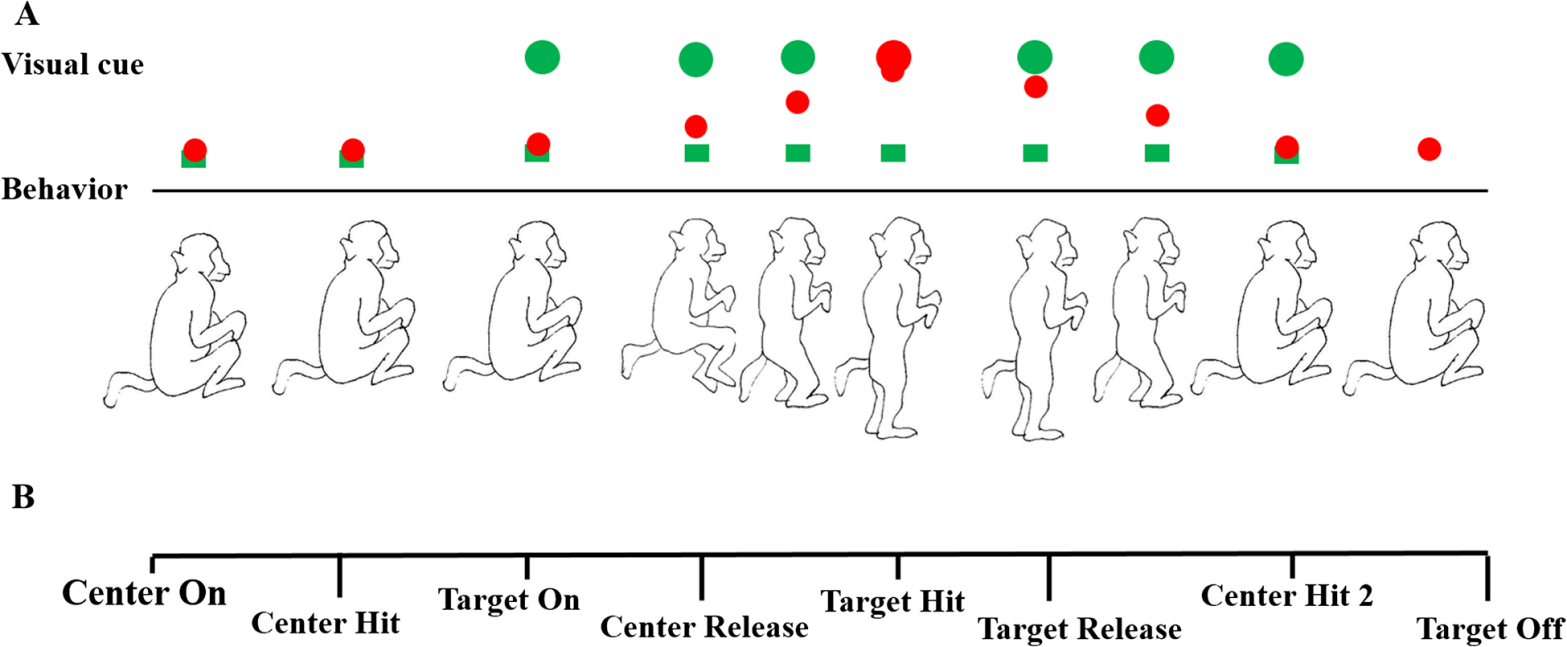
**The behavioural task protocol. A**. Visual cue sequence and behaviour of the monkey in different motion stages. **B**. Events and time intervals in a typical successful trial.

The subjects performed the sit and stand task in a virtual reality environment. Figure 1 showed the procedure of the behaviour task in a typical successful trial. A LED marker placed at the ankle was represented as a red ball in the animation environment. Each trial was preceded by an inter-trial interval (varied randomly from 5 to 10 seconds) when the screen was illuminated with bright blank scene to prevent dark adaptation since the room was in low light condition. A trial was commenced with appearance of a green box representing the starting position (Center On). The subject was required to move his ankle position to match the green box (Center Hit) for 100 msec. After that, another green ball (Target) was displayed on the top of the screen (Target on). The subject was required to push down the pedal to match the ankle cursor with target position. Then the box turned back to green (Center Release). The subject was trained to fully extend his legs to hit the green ball (Target hit) for up to 400 ms and the counterweight matches the body weight to simulate the standing. Then, the subject was required to retract both legs back to the starting position (Target release). When animal fully retracted both legs, the cursor hit the centre box again, and the box turned into red (Center hit again). After another 100 ms, both of the center box and the target ball disappeared indicating the end of a trial (Center and Target off). The subject received his reward. The process entered into another inter-trial interval period. For this experiment, we expected to record kinematics from the legs through the whole stand and sit task.

### Surgery

Under deep general anaesthesia, 3 headpieces were surgically implanted to allow head restraint. At a second surgery, a single 23×20 mm recording chamber was mounted so as to give access to the primary motor area for recordings in the left hemisphere. After the craniotomy surgery had been made, the monkeys received a full course of antibiotics (20 mg/kg oxytetracycline, i.m.) and analgesic (10 μg/kg buprenorphine, i.m).

Separate surgical procedures were performed to implant fine-wire intramuscular electrodes for electromyogram (EMG) into 6 selected lower limb muscles from each leg, including right and left soleus (RS and LS), right and left tibialis anterior (RTA and LTA), right and left semitendinosus (RST and LST), right and left recutus femoris (RRF and LRF), right and left extensor digitorum longus (REDL and LEDL), and right and left flexor digitorum longus (RFDL and LFDL).

### Acute cortical activity and lower-limb electromyogram recordings

Single-unit activities were recorded using a microdrive recording system (Thomas Recording system, Germany). The location of each electrode penetration was determined by triangulation on fiducial markers on the chamber lid, which allowed the stereotaxic position of each new penetration to be calculated. The actual coordination of M1 area in stereotaxic apparatus can be obtained accurately according to the atlas of monkeys’ brain anatomy. Before every penetration, the positions of each electrode were calculated precisely with the method above to ensure that the recording area was within the right range. Lower-limb EMGs were recorded synchronously with a sampling rate of 1000 Hz.

### Signal analysis

Neural signals corresponding to extracellular action potentials were conditioned and amplified, filtered, digitized and stored using a 64-channel neuron recording system (Plexon Inc., Dallas, TX). About 20 successful trials formed a recording session. Spike sorting was performed to isolate individual neuronal unit on each data session based on the clustering of detected action potential waveforms in principal components (PC) feature spaces using the software tool of Offline Sorter (Plexon Inc., Dallas, TX). Sorted data from each session were imported to Neuroexplorer (Plexon Inc.) for statistical analysis. Peri-event histogram analysis was applied to examine patterns of multiunit activity associated with events of lower limb kinematics in motor tasks, with special attention on: initiation of stand, stand up, stand hold on and sit down during the position control task.

A one-way analysis of variance (ANOVA) with p < 0.05 was applied to classify neurons based on their statistical properties of neural activity (firing rate) in each epoch, compared to the average firing rate during the whole trial. The firing rate of each neuron was computed in a 200 ~ 500 milliseconds time window with 30-millisecond bins.

Density contour of neuronal distribution was calculated at each penetration point as the number of neurons related to event divided by the total number of cells recorded under that penetration. Then the densities for all the penetrations were smoothed by a median filter, and the density contour for neuronal distribution related to events was plotted. The hottest colour represented the highest density.

We also investigated the innervating relationship between cortical neuronal units and lower-limb muscles. A multi-input-single output (MISO) linear model was built to depict the correlation of the EMG activities of each muscle in lower limbs using primary motor cortical (M1) neurons. This MISO model utilized the current and historical spiking rates of multiple motor neurons as input to predict the current rectified EMG signal:

1 $$ {\mathrm{y}}_m(t)={\displaystyle \sum_{i=1}^N{\displaystyle \sum_{\tau =0}^T{f}_{im}\left(\tau \right){x}_i\left(t-\tau \right)+\varepsilon }} $$

In (1), each spiking rates input x is convolved with its finite impulse response (FIR) function fim. The subscription im stands for the FIR function of neuron i on muscle m, and ε stands for the residue error, and N is the number of total neuron units involved into the prediction. The values of the FIR function fim need to be estimated from the system inputs and outputs in a train dataset. In model implementation, the spiking rate of each neuron was computed in non-overlapping 20 ms time windows; the EMG signals were detrended, full-wave rectified and then filtered by an order 8 Butterworth low-pass filter with a cut-off frequency of 4 Hz. For each session, we used the first 2/3 trials to train the model, and test it on the remaining 1/3 trials.

## Results

We have recorded 1598 units (689 from Monkey H, 909 from Monkey V) that exhibited activity patterns during the task (see Figure 1A). To further in-depth analyses, we classified the neurons for specific action or event coding for control commands.

### Units for initiation of stand up and their distribution area in M1

**Figure 2:**
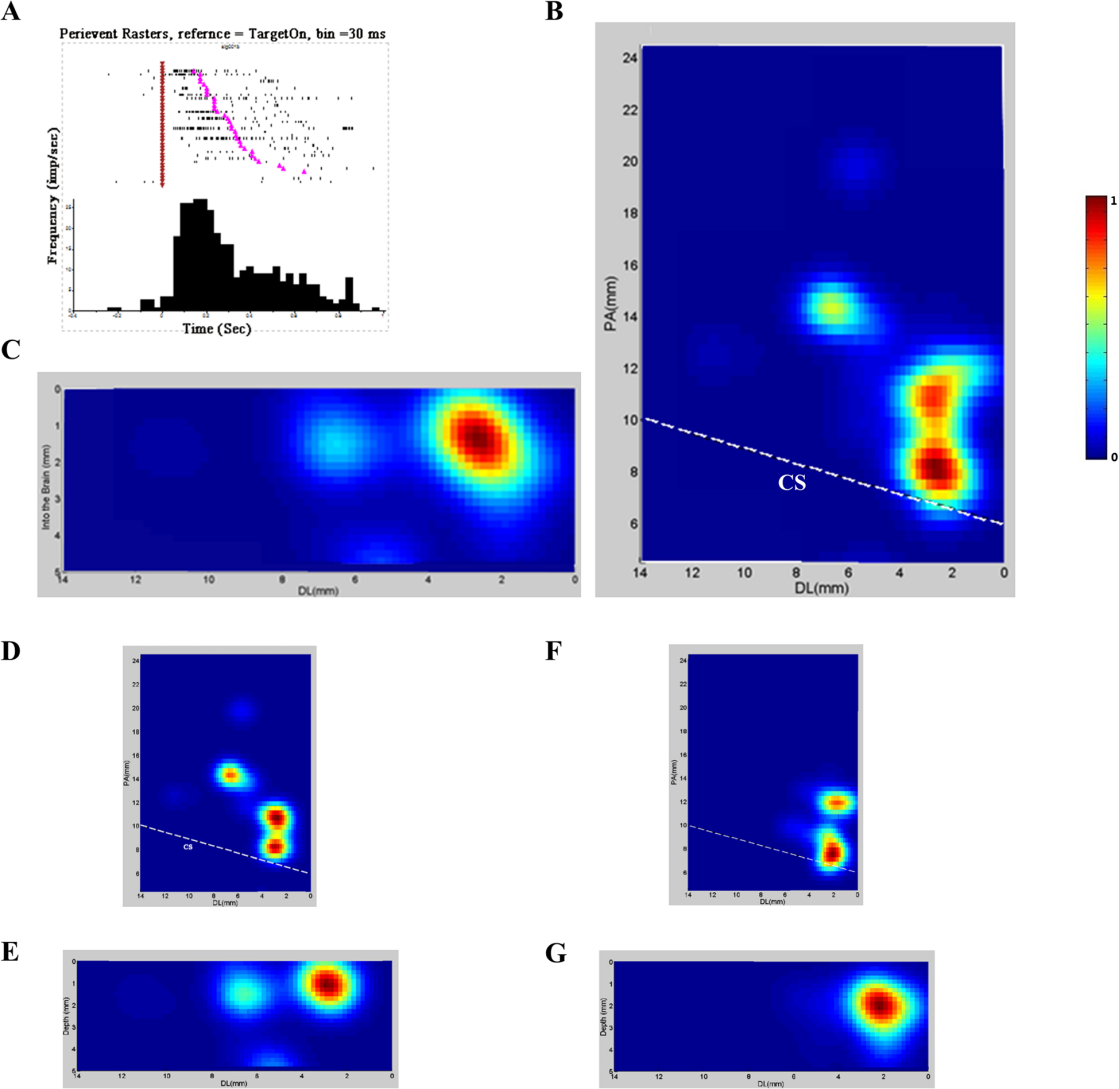
**A sample unit for initiation of stand and the areal distribution of 294 similar units. A**. Peri-event time histogram of an example unit for the initiation of stand-up demonstrated high and tonic activity after target onset (Purple inverted triangle) but inhibited during the actual push and Center Release (Pink triangle), bin size = 30 ms. **B** and **C**. coronal and vertical view of the distribution areas of similar units in two monkeys. **D** and **E**, coronal and vertical view of the distribution areas of similar units in monkey H. **F** and **G**, coronal and vertical view of the distribution areas of similar units in monkey V. These areas are close to the midline (0 mm), just anterior to the central sulcus (dotted white line shown). CS: Central sulcus, PA: posterior–anterior, DL: dorsal-lateral.

When we aligned the data by the command cue (Target on), we found that there were 294 units (169 from Monkey H, and 125 from Monkey V) demonstrated high and tonic activity level after the target on but inhibited during the actual push and Center Release. Figure 2A showed peri-event time histogram (bin size = 30 ms) of an example unit for preparing of stand up, and Figure 2 B and C showed the distribution areas of related units in horizontal and vertical levels. The main area ranged from +7.0 to 12 mm in the Posterior-Anterior dimension, from +0.8 to 3.0 mm in Dorsal-lateral dimension, and from 0.4 to 3.2 mm in depth from brain. Figure 2 D and E showed the distribution areas of related units in horizontal and vertical levels from Monkey H, and 2 F and G, Monkey V. Across the two monkeys and based on standard coordinates, the coordinates of highest densities were almost the same during the initiating of stand.

### Units for stand up and their distribution area in M1

**Figure 3:**
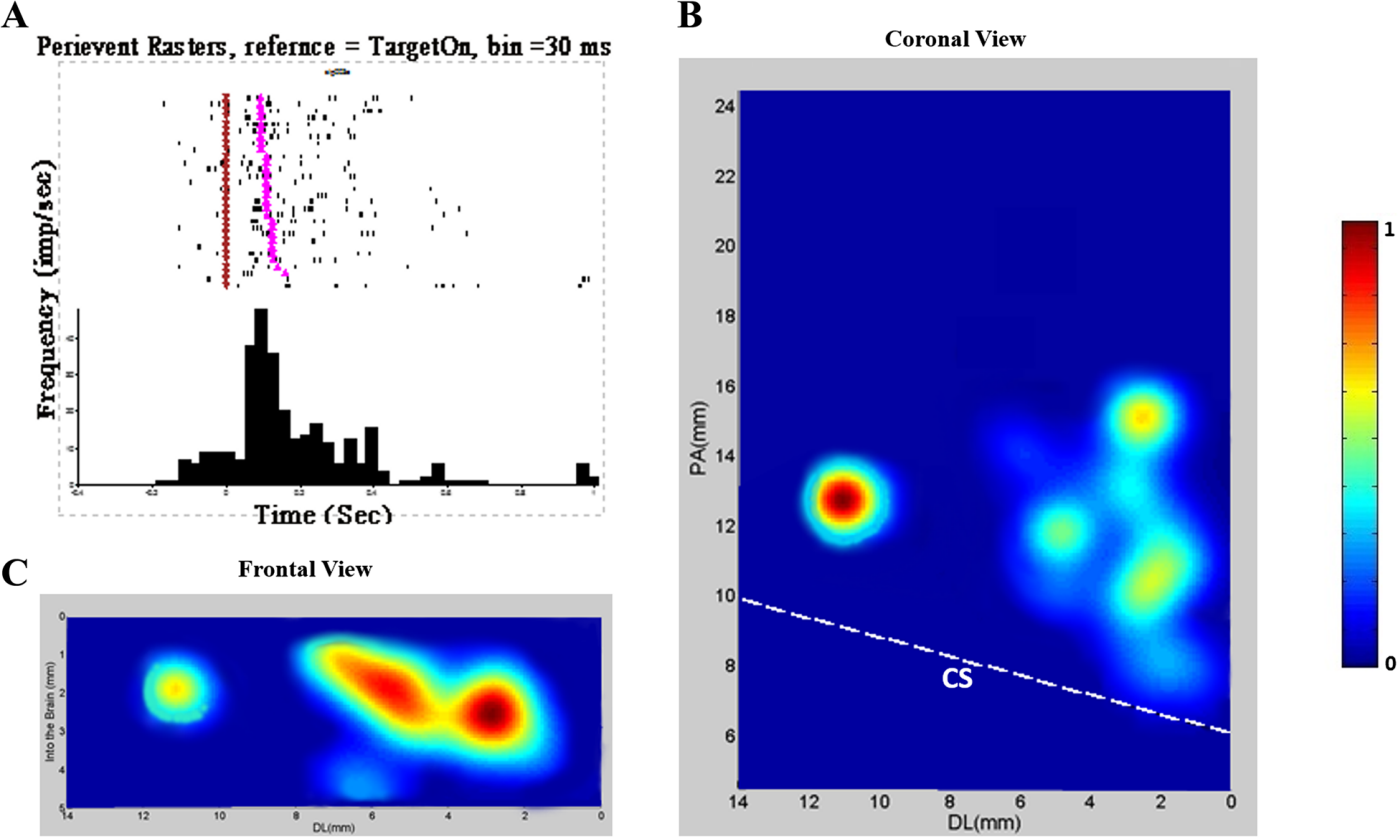
**A sample unit for standing up and the distribution areas of 310 related units.** A. Peri-event time histogram (bin size = 30 ms) of an example unit firing for standing up. Purple inverted triangle: Center Release; pink triangle: Target Hit. B and C. coronal and vertical view of the areal distribution of similar units. CS: Central sulcus, PA: posterior–anterior, DL: dorsal-lateral.

When we aligned the data by the event Center Release, we found that there were 310 units demonstrated high and tonic activity level during pushing phase, i.e. after the Center Release but before Target Hit. Figure 3A showed an example unit for stand up (peri-event time histogram, bin size = 30 ms), and Figure 3B and C showed the distribution areas of the “stand up” units. The distribution area ranged from +6.0 to 16.0 mm in the Posterior-Anterior dimension, from +0.8 to 11.0 mm in Dorsal-Lateral dimension, and from 0.2 to 3.2 mm in depth from brain.

### Units for stand hold and their distribution area in M1

**Figure 4:**
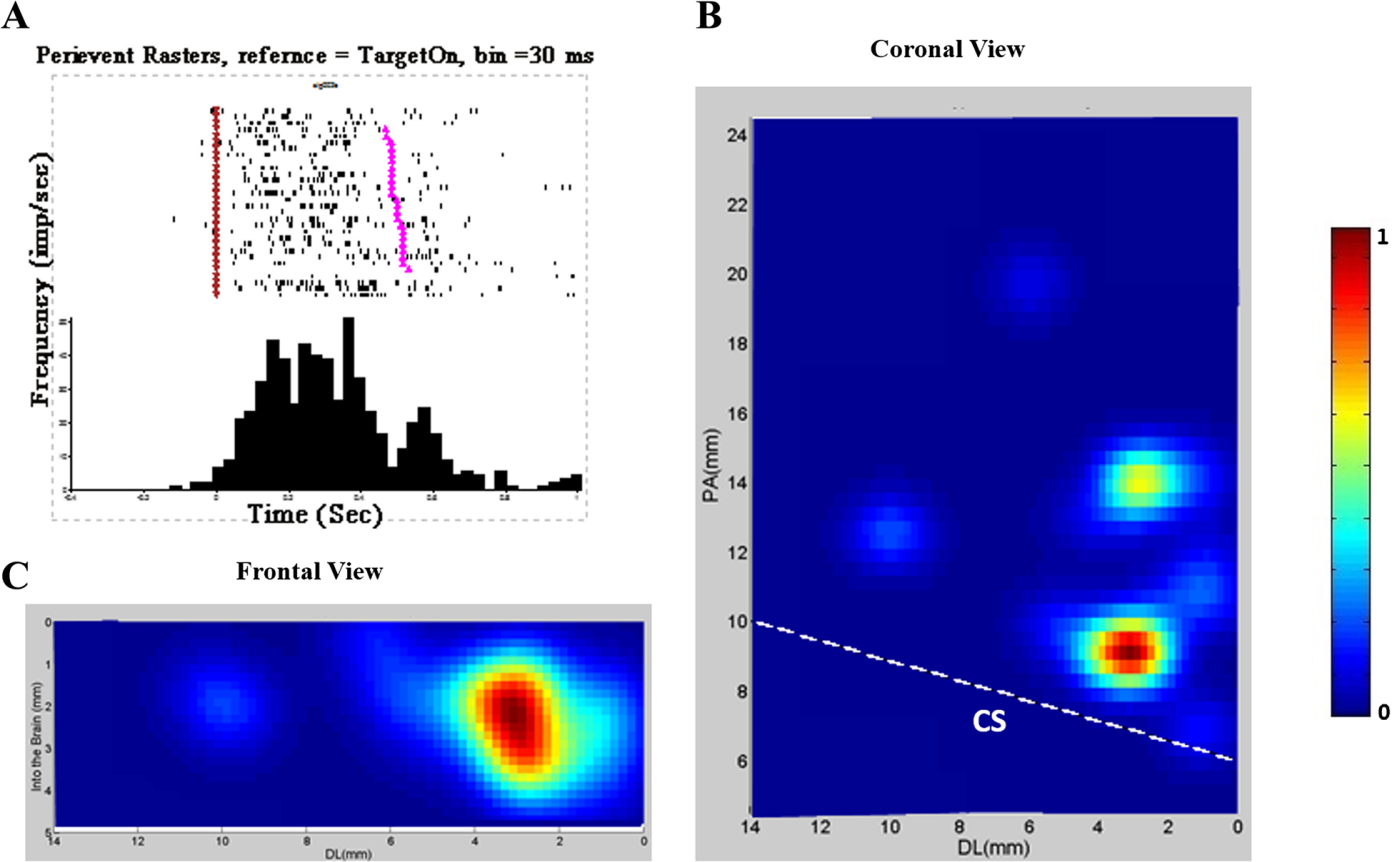
**A sample unit for holding standing and the areal distributions of 104 similar units.** A. Peri-event time histogram (bin size = 30 ms) of an example unit with peak activity during the holding phase. Purple inverted triangle: Target On; pink triangle: Center Hit again. B and C. coronal and vertical view of the areal distribution of related units. CS: Central sulcus, PA: posterior–anterior, DL: dorsal-lateral.

When we aligned the data by the event Target Hit, we found that there were 104 units firing during all holding activities phase, from the Target Hit to Target Release. Figure 4A showed an example unit for stand up (peri-event time histogram, bin size is 30 ms), and Figure 4B and C showed the distribution area of the “stand holding” units. The distribution areas of 104 ranged from +8.0 to 10.0 mm in the Posterior-Anterior dimension, from +2.0 to 4.0 mm in Dorsal-lateral dimension, and from 0.8 to 4.0 mm in depth from brain.

### Units for sit down and their distribution area in M1

**Figure 5:**
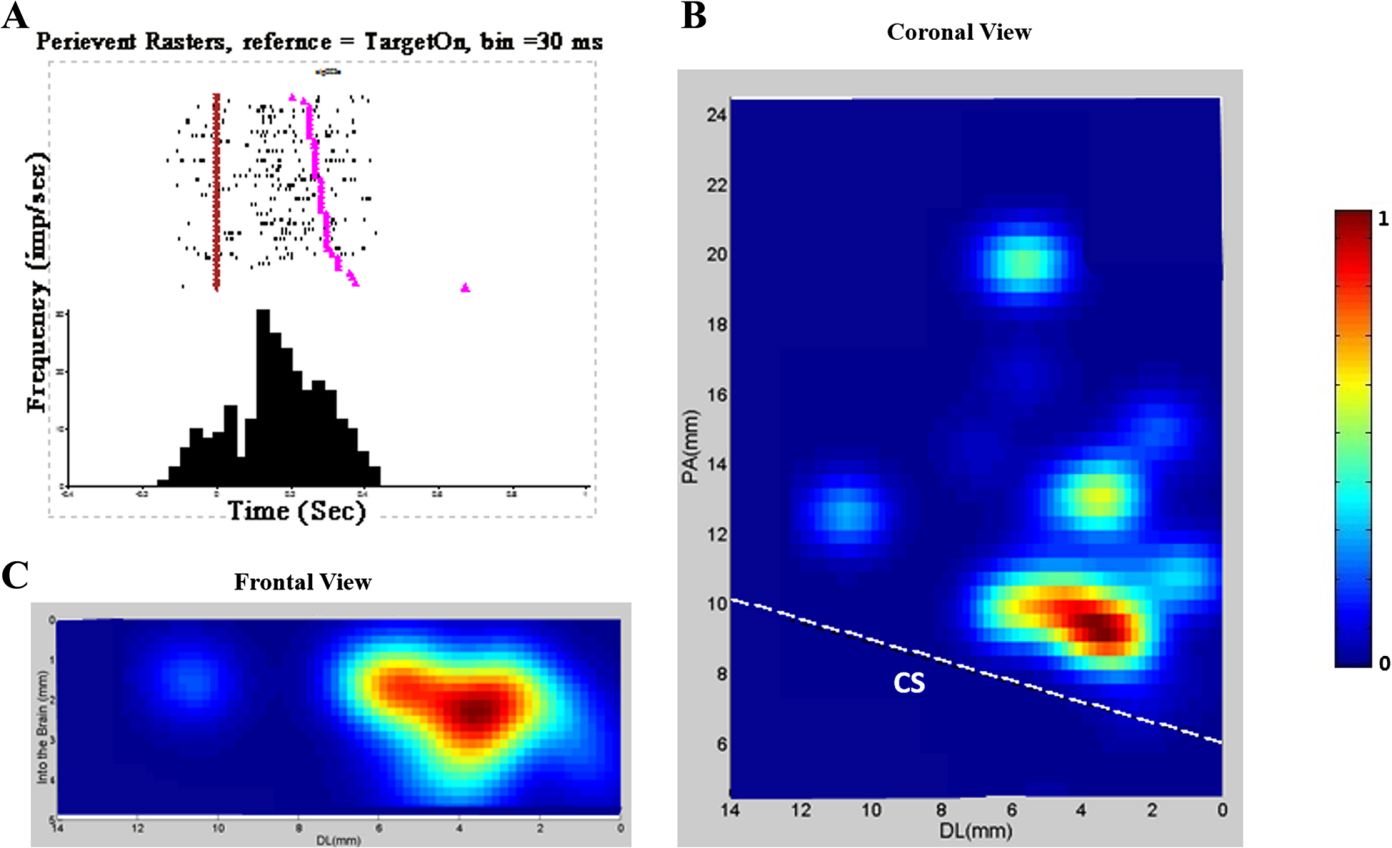
**A sample unit for sitting down and the areal distribution of 205 similar units.** A. Peri-event time histogram (bin size = 30 ms) of an example unit for squatting down showed high peak activity during the leg releasing phase. Purple inverted triangle: Target Release; pink triangle: Center Hit again. B and C. coronal and vertical view of the distribution areas of related units. CS: Central sulcus, PA: posterior–anterior, DL: dorsal-lateral.

When we aligned the data by the event Target Release, we found that there were 205 units demonstrated high and tonic activity level during squatting down phase, after the Target Release but before Center Hit Again. Figure 5A showed an example unit for squatting down (bin size: 30 ms). Figure 5B and C showed the distribution area of the similar units. The distribution areas ranged from +8.0 to 12.0 mm in the Posterior-Anterior dimension, from +2.0 to 6.0 mm in Dorsal-lateral dimension, and from 1.0 to 4.5 mm in depth from brain.

### EMG predictions using event-related neurons

**Figure 6:**
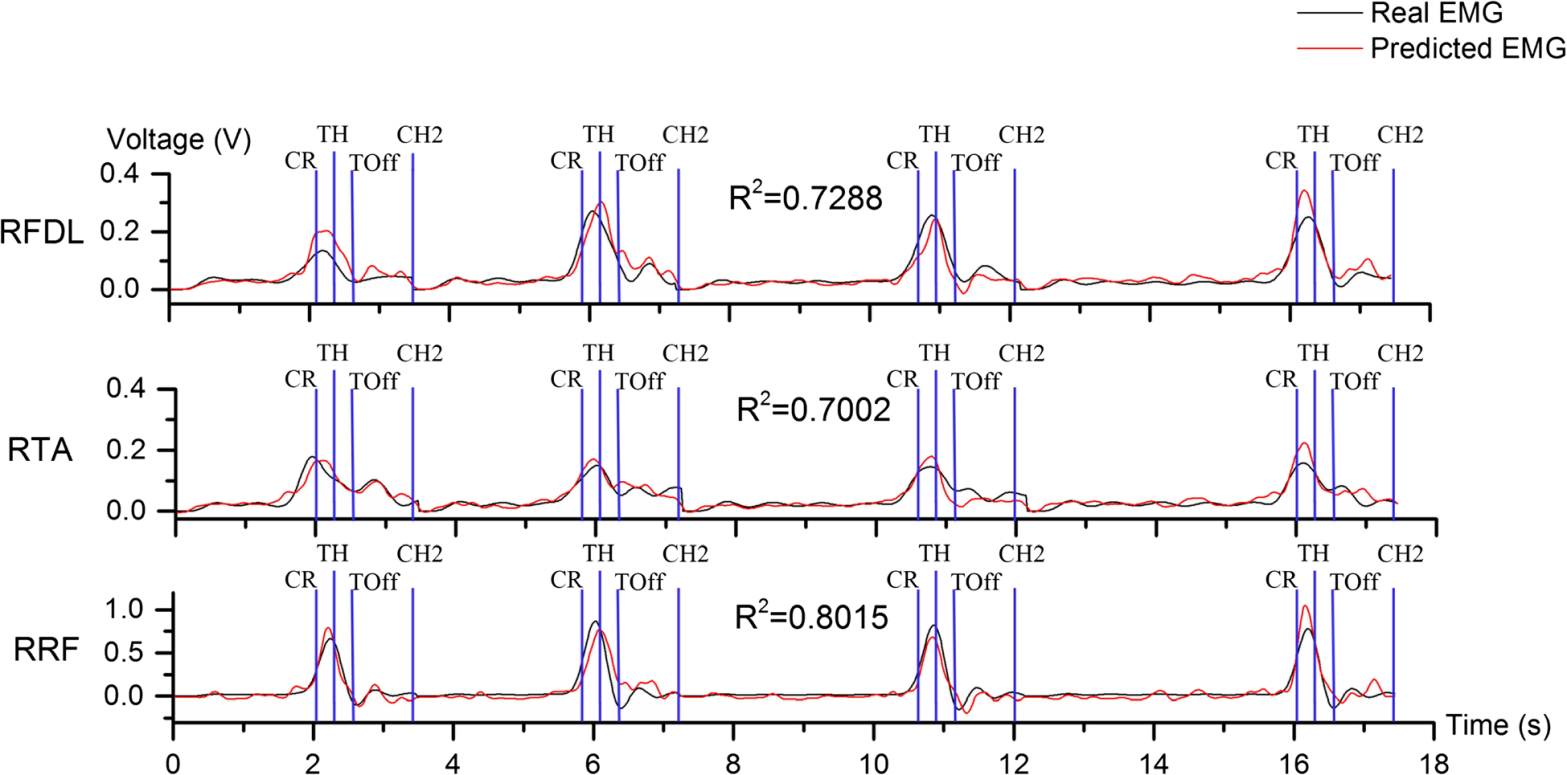
**Real (black) and predicted rectified EMG (red) signals in four successful trials of one session (Monkey H).** Utilizing a multi-input-single output (MISO) linear model, we have predicted EMG activities successfully, with the collected cortical neuronal activities from a small number of neurons in M1. RFDL: right flexor digitorum longus; RTA: right tibialis anterior; RRF: right recutus femoris.

Using the model given as (1), we applied a small number of neurons (8–10) to predict monkey’s lower-limb muscles activities. Figure 6 showed the results of predicting monkey H’s EMG activities of several lower-limb muscles with task-related neurons using the model given as (1). The coefficient of determination (*R*^*2*^) was utilized to evaluate the performance of the model on the testing set. The value of *R*^*2*^ ranged from 0.6 to 0.8 with different neuron-muscle pairs. The results in Figure 6 indicated that predicted EMG given by the linear model in (1) can capture the characteristics of the real EMG accurately.

Table 1 described the percentages of sessions where the R^2^’s are greater than 0.5, and the highest R^2^’s we got in all sessions, for each EMG. We can see that for all 6 hind limb EMG signals, a large portion of sessions has poor predictive performance by the cortical neuronal activities. However, for each lower limb EMG signal, we still harvested quite a few sessions with high R^2^ values.

## Discussion

A fundamental issue which should be addressed in priority was to describe the details of topographic information in M1 related to the lower limb functions. Although the spatial mapping of the leg area in M1 has been repeatedly confirmed in the last decades, using different techniques such as microstimulation or fMRI [35-37], the accurate coordinates of brain area for electrode array implantation was still not very specific.

Constructing multi-electrode arrays based BMI required more accurately parameters.

In this work, we identified the cortical areas related to lower movements in M1 areas by acute neuronal recording methods. We found that all these neurons showed a highly overlapping distribution, which suggested that for practical applications of brain-machine interface, the general information for lower limb movements could be obtained together from the same motor cortical region (Coordination: DL: 0.8-3.6; PA: 9–12; Depth: 1–3).

For the initiation of standing up, the distribution areas from coronal view was relatively small. The hot area ranged from +6.3 to +12.3 mm in the Posterior-Anterior dimension, and from +0.4 to 4.0 mm in Dorsal-lateral dimension. The vertical distribute depths were also relatively narrow, ranged from 0.5 mm to 2.5 mm.

For the standing up, the distribution areas in coronal view got much bigger. Most of the units distributed in the range from +8.0 to 16 mm in the Posterior-Anterior dimension, and from +1.6 to 6.0 mm in Dorsal-lateral dimension. The distribution depths in vertical view were also relatively narrow, ranged from 0.5 mm to 3.0 mm from the brain.

The most interesting was that we still found 104 units related to standing holding, most of them distribution in the range from +8.0 to 14 mm in the Posterior-Anterior dimension, and from +1.5 to 4.5 mm in Dorsal-lateral dimension. The distribution depths in vertical view were also relatively narrow, ranged from 0.5 mm to 4.5 mm. It was generally thought that the brain does not participate standing holding, but we recorded some cortical neurons firing during standing holding. It may be caused by the task which is guided by visual cues, which recruited certain numbers of neurons to perform the task.

For the 205 squatting down related units, most of their distribution was in the range from +8.0 to 14 mm in the Posterior-Anterior dimension, and from +1.5 to 7.0 mm in Dorsal-Lateral dimension. The vertical distribution depths were also relatively narrow, ranged from 0.5 mm to 4.5 mm.

The results about lower-limb motor control here had some spatially overlapping functional maps with that of hand and arm control in reach and grasp task (Figure 2). One of possible reasons for causing such overlapping may lie on the difficulties of excluding the neuronal activity associated with fore-limb movements during the tasks. However, distinctions were observable. Lower-limb areas were much closer to the central sulcus and the midline.

The main aim for constructing a model using task-related neurons’ spiking rates to predict the EMG envelop of each muscle in lower-limb was to investigate whether the innervating relationship between cortical neuronal units and lower-limb muscles existed and can be depicted in some determinate form. The results of figure 6 indicated that a relatively straight forward linear model can convey such neuron-muscular innervation relationship quite well, though it was believed the whole cortical motor control process contained a lot of nonlinearity. The successful application of linear model here suggested that the neurons obtained in our acute recording were highly muscular control related.

Future work will include applying the neuron selection method proposed in this paper on chronically recorded neural datasets and developing on-line decoding algorithms. We will implant multiple microelectrode arrays in the area ranged from +9.0 to 12 mm in the Posterior-Anterior dimension, and from +0.8 to 3.6 mm in Dorsal-lateral dimension, and centre of 2.0 mm in depth from the brain surface. It is very close to the middle line and central sulcus. There are rich of blood vessels over this region, which might easily cause accidentally bleeding during implant surgery. More seriously, most of this area is covered by a large sinusoid on the top of middle line. Unlike blood vessels, the sinusoidal membrane is more easily damaged by sharp tools, and the damage is irreparable. It is almost a taboo to do any surgery in this area if we cannot find a way to escape touching the surface of these tissues. To address this issue, we design a special micro-electrode array for chronic cortical recording. With long length of the electrodes, we could implant the arrays at a big angle from the lateral side so as to escape touching the surface of direct top. And because of implant angle, we design the different length of the two raw electrodes (2.5 mm in one row, and 3.5 mm in another row).

## Conclusions

The present study has examined the neuronal activity related to lower limb voluntary movements in M1 and found various neurons tuned to different stages of the stand and squat task. Utilizing a multi-input-single output (MISO) linear model, we have successfully predicted EMG signals from the small number of collected neurons. Most importantly, we have obtained the topographic information and could locate these neurons related to lower limb activity. This work may contribute to the understanding of the fundamental principles of neural control of lower limb movements and the further development of cortically controlled lower limb neuroprosthetics.
